# 

**Published:** 1896-09-12

**Authors:** 


**Notices of Births, Deaths, and Marriages** are inserted in this column at a charge of 2s. 6d. for an announcement not exceeding four lines.

### NOTICE TO CORRESPONDENTS.

All MS., Letters, Books for Review, and other matters intended for the Editor should be addressed The Editor, The Lodge, Porchester Square, London, W.

The Editor cannot undertake to return rejected MS., even when accompanied by stamped directed envelope.

**Notice to Advertisers and Subscribers.**

All Advertisements, Orders for Copies of The Hospital and Business Communications should be addressed to **THE MANAGER, (Not to The Editor),** The Hospital, 428, Strand, London, W.C.

The Cost of Subscription, post free, is as follows:—Per week, 2½d.; per quarter, 2s. 8d.; per half-year, 5s. 3d.; per annum, 10s. 6d. Foreign:—Per Annum, 15s. 2d.; payable, in all cases, in advance.

Price 5s.
**"Burdett's Hospitals and Charities, 1896."**

Seventh year of publication. Over 1,000 pp. Scarlet cloth, gilt lettered, Price 5s. A most complete guide to British, Colonial, Indian, and American Hospitals, Dispensaries, Nursing and Convalescent Institutions, and Asylums. A few complete sets of the preceding six issues of the "Annual" may still be obtained on application to the Publishers, The Scientific Press (Limited), 428, Strand, London, W.C.

### THE STUDENT OF MEDICINE.

### St. Thomas's Hospital.—House Appointments.

The following gentlemen have been selected as house officers from Tuesday, September 1st, 1896. House physicians: W. H. J. Paterson, L.R.C.P,, M.R.C.S. (extension); E. H. T. Nash, L.R.C.P., M.R.C.S., (extension); G. J. Conford, B.A., M.B., B.Ch.Oxon., L.R.C.P., M.R.C.S., and L. W. Richards, M.B., B.S.Durh., L.R.C.P., M.R.C.S. House surgeons: B. Dyball, L.R.C.P., M.R.C.S.; P. W. Kent, L.R.C.P., M.R.C.S.; J. Smith, M.A., M.B., B.C.Cantab., L.R.C.P., M.R.C.S.; and W. D. Frazer, L.R.C.P., M.R.C.S. Assistant house surgeons: A. J. Martineau, L.R.C.P., M.R.C.S.,; F. H. Gervis, L.R.C.P., M.R.C.S.; R. G. Strange, L.R,C.P., M.R.C.S.; and G. E. O. Taylor, L.R.C.P., M.R.C.S. Obstretric house physicians: (Senior) P. L. Blaber, L.R.C.P., M.R.C.S.; and (junior) E. L. Collis, B.A., M.B., B.Ch.Oxon, L.R.C.P., M.R.C.S. Ophthalmic house surgeons: E. A. Saunders, M.A., M.B., B.Ch.Oxon., L.R.C.P., M.R,C.S.; and P. S. Hitchens, M.A., M.B., B.Ch.Oxon., L.R.C.P., M.R.C.S. Clinical assistants in the special Department for Diseases of the Throat: H. G. Toombs, L.R.C.P., M.R.C.S.; and W. J. Orbell Ray, L.R.C.P., M.R.C.S. Skin: A. H. P. Dawnay, L.R.C.P., M.R.C.S.; and R. Lawson, L.R.C.P., M.R.C.S. Ear: C. E. Durrant, L.R.C.P., M.R.C.S. (extension); and E. O. Thurston, L.R.C.P., M.R.C.S. Clinical assistants in the Electrical Department; W. D. Knocker, L.R.C.P., M.R.C.S. (extension).

### VACANCIES.

The following vacancies are announced this week :—

#### Resident Medical Officers.

Bradford Children's Hospital.—House Surgeon; doubly qualified. Salary £70 per annum, with residence and washing. Applications to C. V. Woodcock, Secretary, by September 14th.

Cheltenham General Hospital.—House Surgeon; unmarried. Must possess a registered qualification in medicine and surgery. Salary £80 per annum, with board and apartments. Applications to H. T. Carrington, Honorary Secretary and Treasurer, by September 19th.

Convalescent Hospital and Sea Bathing Infirmary, Southport.—Resident Medical Officer; unmarried, and not under 27 years of age; doubly qualified. Salary to commence, £150 per annum, with board, lodging, and washing. Applications to the Chairman by September 12th.

Essex and Colchester Hospital.—House Surgeon; doubly qualified. Salary £100 per annum, with board and lodging. Applications to the Committee by September 11th.

Glamorgan County Asylum, Bridgend.—Junior Assistant Medical Officer. Asylum experience essential. Salary £130, rising £10 a year to £150 if approved, with board, lodging, and washing. Applications to the Medical Superintendent by September 16th.

Hants County Asylum.—Assistant Medical Officer; unmarried; doubly qualified and registered under the medical Act. Candidates should not exceed the age of 25 years. Salary £100 per annum, increasing to £125 after twelve months' service, with apartments, board, washing, and attendance. Applications forwarded by September 21st, addressed to the Committee of Visitors, Knowle, Fareham, endorsed "Application for appointment of Medical Officer."

London Temperance Hospital. Hampstead Road, N.W.—Assistant Resident Medical Officer; doubly qualified. Appointment for six months. No salary attached to the post, but board, washing, and residence in the hospital provided. Applications to A. W. Bodger, Secretary, by September 16th.

Manchester Southern and Maternity Hospital.—Resident House Surgeon. Honorarium at the rate of £30 per annum, and board. Applications to George William Fox, 53, Princess Road, by September 12th.

Royal Free Hospital, Gray's Inn Road, W.C.—Two House Physicians. Appointments for six months. No salary, but board, &c., provided. Applications to the Secretary by September 21st.

Victoria Hospital for Sick Children, Queen's Road, Chelsea, S.W.—House-Surgeon; must be F. or M.R.C.S.Eng., and L.S.A. Honorarium of £50 per annum, with board and lodging in the hospital. Appointment for twelve months. Applications to Commander Blount, R.N., Secretary, by September 12th.

#### Non-Resident Medical Officers.

Hampstead Hospital.—Vacancy in the Staff. Candidates must be legally registered, and possess Medical and Surgical qualification. Applications to R. A. Owthwaite, Honorary Secretary, by September 14th.

Owens College, Manchester.—Senior and Junior Demonstrator in Physiology. The stipends £150 rising to £200, and £100 rising to £150 respectively. Applications to S. Chaffers, Registrar, by September 22nd.

